# A Selective Ensemble Classification Method Combining Mammography Images with Ultrasound Images for Breast Cancer Diagnosis

**DOI:** 10.1155/2017/4896386

**Published:** 2017-06-27

**Authors:** Jinyu Cong, Benzheng Wei, Yunlong He, Yilong Yin, Yuanjie Zheng

**Affiliations:** ^1^School of Information Science and Engineering, Key Lab of Intelligent Computing & Information Security in Universities of Shandong, Institute of Life Sciences, Shandong Provincial Key Laboratory for Distributed Computer Software Novel Technology, and Key Lab of Intelligent Information Processing, Shandong Normal University, Jinan 250358, China; ^2^College of Science and Technology, Shandong University of Traditional Chinese Medicine, Jinan 250014, China; ^3^School of Computer Science and Technology, Shandong University, Jinan 250100, China

## Abstract

Breast cancer has been one of the main diseases that threatens women's life. Early detection and diagnosis of breast cancer play an important role in reducing mortality of breast cancer. In this paper, we propose a selective ensemble method integrated with the KNN, SVM, and Naive Bayes to diagnose the breast cancer combining ultrasound images with mammography images. Our experimental results have shown that the selective classification method with an accuracy of 88.73% and sensitivity of 97.06% is efficient for breast cancer diagnosis. And indicator *R* presents a new way to choose the base classifier for ensemble learning.

## 1. Introduction

Breast cancer is one of the most common malignant tumors in female [1]; it has been the first leading of death among tumors in patients under the age of 45 years old in both Asian and European countries [2]. Early detection and diagnosis of breast cancer play an important role in reducing mortality rates of breast cancer, which can improve the cure rate, relieve patients' sufferings, and guarantee the patients' life quality [3, 4].

Mammography and ultrasound are two most commonly used screening modalities for early detection and treatment of breast cancer. Mammography uses X-ray to noninvasively image the internal structure of human breast, which can be used to show the masses, calcifications, and any other suspicious area that could be identified as signs of breast cancer [5]. However, screening with mammography alone is limited in its ability to detect tumors in dense breasts which is typically linked to a higher risk of cancer [6]. As a supplement modality to mammography, ultrasound has been proven to depict the shape, border, and internal echo features of small, node-negative breast tumors clearly [7]. Meanwhile, the low cost [8] and better performance in dense breast [9, 10] improve the application of ultrasound images in breast cancer diagnosis. In particular for the Asian women, who are with higher density breast tissue, the breast cancer diagnosis based on the mammography has certain limitations. It has been proven that using ultrasound in conjunction with mammography resulted in significantly increased cancer detection rates [11–14].

Moreover, the radiologist's clinical experience and subjective judgment directly affect the accuracy of the diagnosis; the radiologist who lacks clinical experience may make an inaccurate diagnosis or miss a diagnosis. At the same time, the inherent high frequency noise and the shadow of medical images may also affect the accuracy of diagnosis. Therefore, a computer-aided diagnosis (CAD) using ultrasound with mammography which can improve the accuracy and specificity is needed in clinical application [15]. The selective ensemble learning [16] is mentioned for the fusion of the ultrasound and mammography in an efficient way. Compared with traditional ensemble learning, the selective ensemble learning can generate the base classifiers with stronger generalization ability. And it is also have the smaller size and faster speed.

In this paper, we propose a selective ensemble classification method using mammography with ultrasound for breast cancer diagnosis. First, instead of the BI-RAD feature, we extract the texture and morphological features to obtain more information of the lesion's edge, shape, and texture on ultrasound and mammography. Then three classifiers, KNN, SVM, and Navies Bayes, which have been used to diagnose breast cancer by many researchers are employed for the ultrasound and mammography, respectively. To ensure the accuracy and the generalization of the base classifier, the new indicator *R* is proposed to choose the appropriate base classifier. Finally, the results of breast cancer diagnosis is obtained by the integrated classifier by majority vote. The extensive experimental results show that the ensemble classification method is efficient for breast cancer diagnosis in our database. The indicator *R* can choose the base classifiers with high accuracy and generalization.

The rest of the paper is organized as follows. In Section 2, we explain our method. The indicators are mentioned in Section 3. Experimental results of diagnosis are explained in Section 4, and, finally in Section 5, we conclude our work and give the prospect of the future work.

## 2. Method

**Figure 1:**
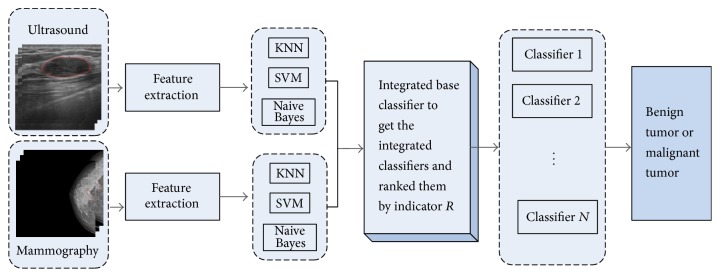
The flow chart of the selective ensemble method.

In this paper, we propose a selective ensemble classification method combining mammography with ultrasound images to diagnose breast cancer. Extracting the feature on ultrasound images and mammography images, respectively, we sampled these images with 10-fold cross-validation to train the base classifiers (SVM, KNN and Naive Bayes for mammography and ultrasound features). Considering that integrating many of the classifiers may be better than integrating all of them, we obtain different integrated classifiers generated by three of all base classifiers and rank them by the indicator *R*. We choose the best integrated classifier and obtain the final result by majority vote to diagnose the breast cancer. The flow chart of the selective ensemble method has been shown in Figure 1.

### 2.1. Feature Extraction

In this paper, we extract the minimum bounding rectangle around the lesion on ultrasound images and mammography images to calculate the gray-level cooccurrence matrix (GLCM) which can provide a method for generating texture features. GLCM are computed in four directions (0°, 45°, 90°, and 135°) with 8-pixel distance. The feature used for classification are: correlation, contrast, energy and entropy. Morphological features are beneficial to distinguishing between benign and malignant tumors on ultrasound images, so we extract three morphological features: depth-to-width (*D* : *W*) ratio, elliptic-normalized circumference (ENC), and the size of the lesion [17]. The *D* : *W* is the ratio of the depth and the width of the minimal circumscribed rectangle of the lesion. ENC is defined as the circumference ratio of the lesion and its equivalent ellipse, and it represents the irregular boundary of the lesions. The benign tumors will stop growing, while the malignant will not stop growing. The larger size is always with the malignant tumor.

### 2.2. The Selective Ensemble Classification Method Combining Mammography Images with Ultrasound Images

As is well known, the integrated classifier may be better than the single classifier. So we propose a selective ensemble classification method combining mammography images with ultrasound images to diagnose breast cancer. We choose the KNN, SVM, and Naive Bayes as the base classifier because they are implemented simply and diagnose breast cancer effectively. The key in ensemble learning is the accuracy and diversity of base classifiers. We use the indicator *R* to rank the single classifier which is based on the accuracy and its diversity. *R* can be described as follows:

(1) $$\begin{array}{c} R_{i}=\mu_{1}*ACC_{i}-\mu_{2}*DF_{i}. \end{array}$$

ACC_*i*_ is the accuracy of the single classifier; double fault (DF) can reflect the diversity of the classifier [18]. As is show in Table 1, *N*^*mn*^ means the number of different classifier's results with *m* and *n* (*m* and *n* are 0 or 1). The smaller *R* is, the better the diversity the classifier will be. The different *μ* can impact the choice of the classifier (*μ*_1_ = 0.4, *μ*_2_ = 0.6):

(2) DFi,k=N00N11+N10+N01+N00,DFi=∑2LDFi,kL−1.

## 3. Indicators

We use six indicators to evaluate the performance of the system, accuracy, sensitivity, specificity, negative predictive value (NPV), positive predictive value (PPV), and the area under receiver operating characteristic curve (AUC).

The accuracy is described as the ratio of the correct samples distinguished by the classifier to the total samples. The accuracy can be described as follows:

(3) $$\begin{array}{c} Accuracy=\frac{TP+TN}{TP+TN+FP+FN}*100\%, \end{array}$$

where the mean of TP, TN, FP, and FN can be seen in the Table 2.

The sensitivity is described as the ratio of the malignancy distinguished by the integrated classifier (TP) to the real malignancy (TP + FN). It can measure that how many times malignancy can be diagnosed. The specificity is described as the ratio of the benign tumor distinguished by the integrated classifier (TN) to the real benign tumor (TN + FP). It measures that how many times benign tumors was misdiagnosed as malignancy. The NPV can measure the diagnosis accuracy of benign tumor. The PPV can measure the diagnosis accuracy of malignancy tumor. These four indicators can be described as follows:

(4) Sensitivy=TPTP+FN∗100%,Specifity=TNTN+FP∗100%,PPV=TPTP+FP∗100%,NPV=TNTN+FN∗100%.

In addition to the above, we also use the AUC which means the area of the ROC (receiver operating characteristic) curve to appraise the performance.

## 4. Experiments

To evaluate the effectiveness of our method, we do some experiments using the indicators mentioned above. The database used in this paper contains 142 medical images, including 71 ultrasound images and 71 mammography images. These images are acquired by 71 patients, 40 benign tumors and 31 malignant tumors. The programs are implemented with Matlab R2010b and WEKA, and data was entered into the computer on Intel 2.93 G dual-core processor with 3.29 G RAM.

### 4.1. The Performance of Single Classifier Compared with the Integrated Classifiers in Ultrasound Images and Mammography Images, Respectively

First, we compared the performance of single classifier with the integrated classifiers based on ultrasound images or mammography images, respectively. As is shown in Table 3, the performance of integrated classifiers is better than the Naive Bayes and KNN on ultrasound images. Compared with SVM, it is better at accuracy, specificity, and PPV, while it is little worse at sensitivity, NPV, and AUC. The integrated classifier and the SVM tend to have different strengths on ultrasound images.

**Figure 2:**
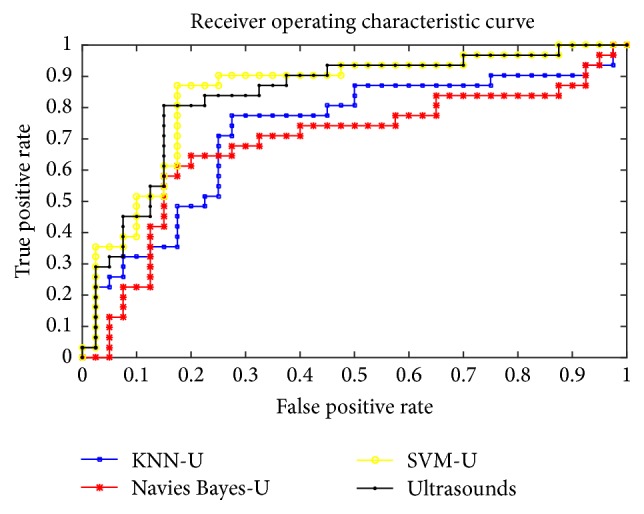
The ROC of the KNN, SVM, and Naive Bayes compared with the integrated classifiers on ultrasound images.

**Figure 3:**
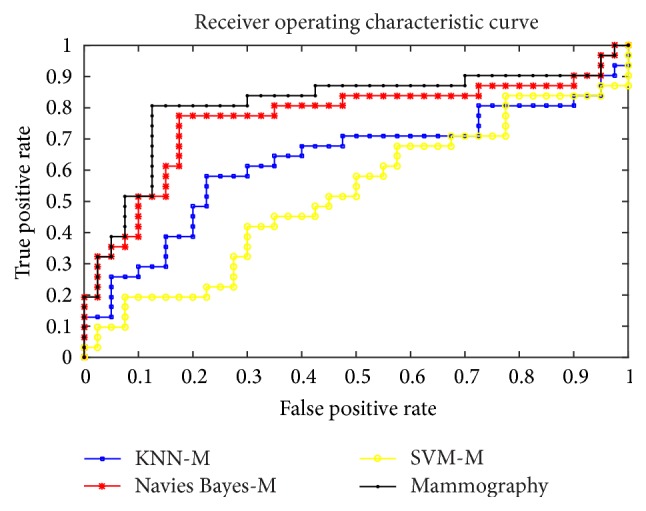
The ROC of the KNN, SVM, and Naive Bayes compared with the integrated classifiers on mammography images.

In Table 4, the single classifier has poor performance on all indicators compared with integrated classifiers on mammography images. (Classifier-M means the results of the mammography images using this classifier. And Classifier-U means the results of the ultrasound images using this classifier. For example, SVM-M means the results of the mammography images using SVM.) The ROC is shown in the Figures 2 and 3. Obviously, the integrated classifier is better than the single classifier.

### 4.2. The Effectiveness and Necessity of Combining the Ultrasound Images with Mammography Images

**Figure 4:**
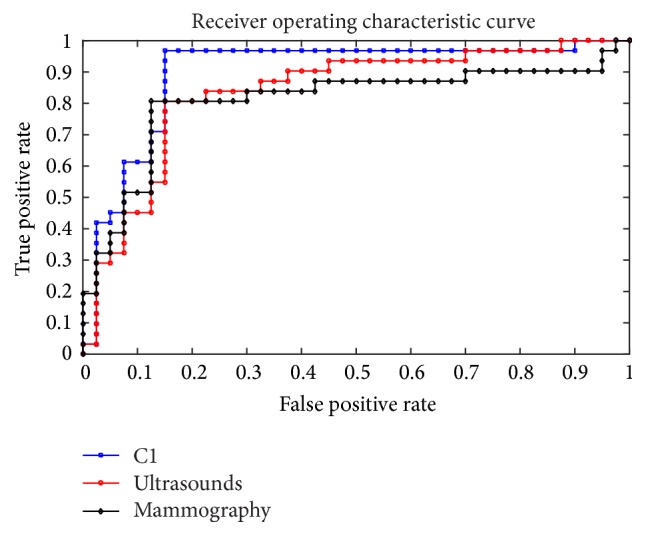
The ROC of the integrated classifier on ultrasound images or mammography images compared with the integrated classifiers on multimodal images.

We propose the ensemble method based on multimodal images (mammography and ultrasound images) according to its complementary feature. In Table 5 and Figure 4, we compared the performance on ultrasound images and mammography images with multimodal images to prove the effectiveness and necessity. The performance on multimodal images is superior to that of mammography images. It is worse than the performance in ultrasound images only in specificity and PPV, but it is superior in accuracy, sensitivity, NPV, and AUC. Typically, the ensemble method can obtain the sensitivity of 96.77%. It is much higher than the integrated classifier on ultrasound images. The higher the sensitivity is, the more the malignancy can be diagnosed. It benefits to early detect breast cancer, so the system with higher sensitivity is valuable in clinical application. So the ensemble method combining the mammography images with ultrasound images has better performance. It is necessary and effective to diagnose breast cancer on multimodal images.

### 4.3. The Different Selection of Base Classifier

**Figure 5:**
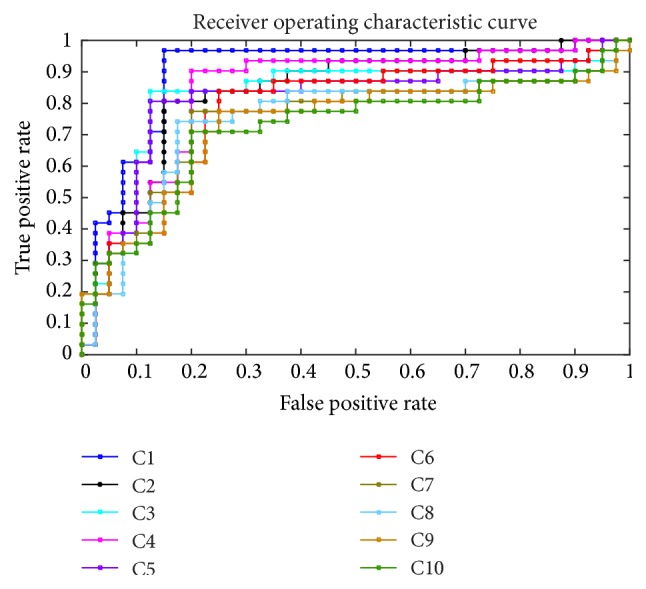
The ROC of the different selection of classifiers.

We have proved that the integrated classifiers are better than single classifier. However, there are so many ways to integrate. We use the indicator *R* to select the base classifier in our work. We ranked the different integrated classifiers with *R* and chose the top 10 integrated classifiers (C1, C2,…, C10). Then we tested their performance to demonstrate the effectiveness of indicator *R*. The performance of the different integrated classifier is shown in Table 6 and Figure 5. We can see that the best value of accuracy, sensitivity, specificity, NPV, PPV, and AUC appeared primarily in the integrated classifiers C1 and C2. So indicator *R* can effectively choose base classifiers. Considering that the sensitivity is more important in the clinical application and the integrated classifier C1 performs much better than the integrated classifier C2 in accuracy and sensitivity, we choose integrated classifier C1 which is ranked first by indicator *R* to diagnose breast cancer.

### 4.4. The Performance of the Classifier-Fusion Method Compared with the Feature-Fusion Method

**Figure 6:**
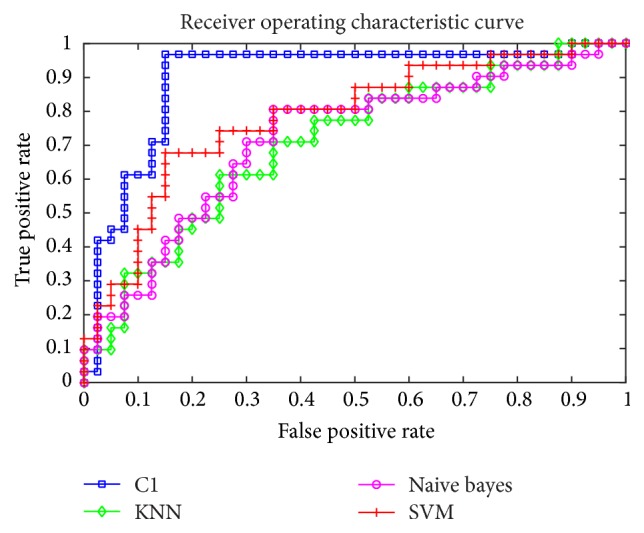
The ROC of the classifier-fusion method compared with the feature-fusion method.

In this paper, we integrated the classifier instead of integrating the feature to prevent the characteristic redundancy. To test the efficiency, we compared it with the different fusion methods. As is shown in Table 7 and Figure 6, the classifier-fusion method is better than the feature-fusion method in all indicators.

### 4.5. The Performance of Our Method Compared with GASEN

GASEN (Genetic Algorithm based Selected Ensemble) [16] is the first selective ensemble learning method proposed by Zhou et al. To evaluate the effectiveness of our method, we compared the performance of our method with GASEN. As is shown in Table 8, our method is better than GASEN in all indicators. The proposed selective ensemble method in this paper is more suitable for breast cancer diagnosis rather than GASEN.

## 5. Conclusion

In this paper, we propose a new selective ensemble method to diagnose breast cancer by combining ultrasound images with mammography images. When generating the integrated classifier, we choose the suitable base classifier using new indicator *R*. Then we test our method in a multimodal database containing 71 breast ultrasound images and 71 mammography images. The selective ensemble method is efficient in diagnosing the breast cancer; we can obtain an accuracy of 88.73% and sensitivity of 97.06%. We also prove that the classifier-fusion method is better than the feature-fusion method in all indicators in our database. In our work, we just choose the simple feature. We will concentrate on the relationship of features ultrasound and mammography images in the future.

## Figures and Tables

**Table 1:** The definition of *N*^*mn*^.

	*D*_*k*_ correct (1)	*D*_*k*_ wrong (0)
*D*_*i*_ correct (1)	*N* ^11^	*N* ^10^
*D*_*i*_ wrong (0)	*N* ^01^	*N* ^00^

**Table 2:** The mean of TP, TN, FP, and FN.

		Actual	
		Positive (malign)	Negative (benign)
Prediction	Positive (malign)	True positive (TP)	False positive (FP)
	Negative (benign)	False negative (FN)	True negative (TN)

**Table 3:** The performance of single classifier compared with the integrated classifiers on ultrasound images.

	Accuracy	Sensitivity	Specificity	NPV	PPV	AUC
Naive Bayes-U	71.83%	54.84%	85%	70.83%	73.91%	0.6831
SVM-U	84.51%	**87.10**%	82.50%	**89.19**%	79.41%	**0.8427**
KNN-U	73.24%	77.42%	70%	80%	66.67%	0.7250
The integrated classifier (ultrasounds)	**85.92**%	83.87%	**87.50**%	87.50%	**83.87**%	0.8363

**Table 4:** The performance of single classifier compared with the integrated classifiers on mammography images.

	Accuracy	Sensitivity	Specificity	NPV	PPV	AUC
Naive Bayes-M	78.87%	77.42%	80%	82.05%	75.00%	0.7653
SVM-M	54.93%	45.16%	62.5%	59.52%	48.28%	0.5202
KNN-M	67.61%	58.06%	75%	69.77%	64.29%	0.6290
The integrated classifier (mammography)	**83.10**%	**80.05**%	**85**%	**85**%	**80.65**%	**0.8089**

**Table 5:** The effectiveness and necessary of multimodal images.

	Accuracy	Sensitivity	Specificity	NPV	PPV	AUC
The integrated classifier based on multimodal images (C1)	**88.73**%	**96.77**%	82.50%	**97.06**%	81.08%	**0.8968**
The integrated classifier on ultrasound images	85.92%	83.87%	**87.50**%	87.50%	**83.87**%	0.8363
The integrated classifier on mammography images	83.10%	80.05%	85%	85%	80.65%	0.8089

**Table 6:** The performance of the different selection of classifiers.

	Accuracy	Sensitivity	Specificity	NPV	PPV	AUC
C1 = Naive Bayes-M + KNN-U + SVM-U	**88.73**%	**96.77**%	82.50%	**97.06**%	81.08%	**0.8968**

C2 = Naive Bayes-U + KNN-U + SVM-U	85.92%	83.87%	**87.50**%	87.50%	**83.87**%	0.8363

C3 = KNN-U + Naive Bayes-U + Naive Bayes-M	84.51%	83.87%	85%	87.18%	81.25%	0.8242

C4 = KNN-U + SVM-U + KNN-M	83.10%	90.32%	77.50%	91.18%	75.68%	0.8363

C5 = Naïve Bayes-U + SVM-U + Naive Bayes-M	84.51%	80.65%	**87.50**%	85.37%	83.33%	0.8097

C6 = KNN-U + SVM-U + SVM-M	77.46%	83.87%	72.50%	85.29%	70.27%	0.7782

C7 = KNN-U + Naive Bayes-M + KNN-M	77.46%	77.42%	77.50%	81.58%	72.73%	0.7500

C8 = KNN-U + Naive Bayes-U + KNN-M	77.46%	74.19%	80%	80%	74.19%	0.7468

C9 = KNN-U + Naive Bayes-M + SVM-M;	74.65%	77.42%	72.50%	80.56%	68.57%	0.7306

C10 = SVM-U + Naive Bayes-M + KNN-M	74.65%	70.97%	77.50%	77.50%	70.97%	0.7234

**Table 7:** The performance of the classifier-fusion method compared with the feature-fusion method.

	Accuracy	Sensitivity	Specificity	NPV	PPV	AUC
Naive Bayes-feature-fusion	69.01%	80.65	60%	80%	60.98%	0.6919
SVM-feature -fusion	76.06%	64.52	85%	75.56%	76.92%	0.7290
KNN-feature -fusion	67.61%	61.29	72.50%	70.73%	63.33%	0.6879
The classifier- fusion method (C1)	**88.73**%	**96.77**%	**82.50**%	**97.06**%	**81.08**%	**0.8968**

**Table 8:** The performance of the our method compared with GASEN.

	Accuracy	Sensitivity	Specificity	NPV	PPV	AUC
GASEN	69.01%	80.65	60	80%	60.98%	0.6919
Our method	**88.73**%	**96.77**%	**82.50**%	**97.06**%	**81.08**%	**0.8968**
